# Thyroid storm-induced acute liver dysfunction and portal hypertension in a patient with Graves' disease: a case report

**DOI:** 10.2144/fsoa-2023-0180

**Published:** 2024-05-14

**Authors:** Mouna Medhioub, Amal Khsiba, Moufida Mahmoudi, Asma Ben Mohamed, Manel Yakoubi, Emna Haouet, Lamine Hamzaoui

**Affiliations:** 1 Université Tunis El Manar, faculté de médecine de Tunis, 1007, Tunis, Tunisia; 2 Gastroenterology department, Mohamed Taher Maamouri Hospital, 8000, Mrezgua, Nabeul, Tunisia; 3 Endocrinology department B, Nationale Institute of Nutrition, 1007, Tunis, Tunisia

**Keywords:** ascites, Graves' disease, hepatic failure, portal hypertension, thyroid storm, thyrotoxicosis

## Abstract

Thyroid storm is a life-threatening condition associated with multiorgan dysfunction and decompensation. We report the case of a 41-year-old woman having Graves' disease presented with thyroid storm complicated with liver dysfunction and portal hypertension in the absence of congestive heart failure or known liver disease. After successful therapeutic management, all biological, clinical and morphological abnormalities regressed.

## Plain language summary

Graves' disease is an immune system disorder that results in the overproduction of thyroid hormones. We report the case of a 41-year-old woman with Graves' disease who presented with reversible liver dysfunction and high blood pressure in the portal vein (located in the abdomen) following the withdrawal of her treatment.

## Abstract (plain language summary)

Thyroid storm is is an endocrinological emergency.

Diagnosis is based on clinical data in a patient with underlying hyperthyroidism.

Hepatic dysfunction is uncommon in the absence of congestive heart failure or known liver disease.

Reversible portal hypertension is an exceptional complication of thyroid storm.

Thyroid storm or thyrotoxic crisis is a rare life-threatening condition that, usually, complicates underlying Graves' hyperthyroidism [1]. Clinical manifestations include exacerbation of hyperthyroidism's symptoms and multivisceral dysfunction/decompensation involving: cardiovascular system, central nervous system, gastrointestinal tract, liver and thermoregulation [2]. Thyroid storm is a medical emergency as it is associated with an increased risk of mortality up to 10–30% of cases [3,4]. Hepatic dysfunction in thyroid storm range from deranged liver function tests to acute liver failure. We report the case of thyroid storm complicated by hepatic dysfunction and portal hypertension.

## Case report

A 41-year-old woman with a history of Grave's disease of 4 years' standing was admitted to the endocrinology department with a 3-month history of asthenia, abdominal distension and dark urine and clay-colored stools. She was treated with antithyroid drug (Benzylthiouracil 125 mg per day) and beta blockers (propanolol). Thyroid function was last checked 4 months ago, showing a slight increase in FT4 levels (FT4 = 20 pmol/l). The patient stopped her treatment of her own will, one month before the hospitalization. She had a history of type I diabetes with ketosis-onset of 6 months' standing, treated with insulin and characterized by frequent hypoglycemic attacks. In the previous year, a uterine myoma was revealed by menorrhagia associated with severe anemia requiring blood transfusions.

On physical examination, the patient was agitated, febrile at 38.9 with profuse sweating. Thyroid examination revealed a goiter with moderate exophthalmos. Cutaneous and mucosal examination revealed icterus with melanoderma of the face and hyperpigmentation of the palmar creases. On cardio-vascular examination, the patient presented with tachycardia at 130 bpm, orthostatic hypotension (blood pressure in the upright position = 15/9 mmHg and in the supine position = 11/7 mmHg) with edema of lower limbs without signs of right heart failure. The respiratory rate was 24 breaths per min and the cardiopulmonary auscultation was normal. The abdominal examination revealed abdominal distension with abundant ascites. Laboratory data (Table 1) showed a normochromic microcytic anemia, thrombopenia, hyperbilirubinemia without cholestasis or cytolysis. The prothrombin time was low (53%) with an INR 1.3. We also noted hypocholesterolemia and hypoalbuminemia. Renal function and blood electrolytes were normal. There was no biological inflammatory syndrome. Peritoneal fluid analysis revealed exudative pauci cellular ascitic fluid (albumin = 30 g/l, serum ascites albumin gradient <11).

**Table 1.** Laboratory investigations of the patient.

Laboratory parameter	Value during hospitalization	Value after 3 months	Normal range
FT4 (pmol/l)	76	15.8	7–17
Hb (g/dl) WBC (E/mm^3^) Plt (E/mm^3^)	6.9 4100 67,000	12.2 5800 145,000	12–17 4000–9000 150,000–400,000
AST (UI/l) ALT (UI/l) T-Bili (μmol/l) D-Bili (μmol/l) ALP (UI/l) GGT (UI/l)	25 38 63 46 64 24	22 29 14 4 71 30	<40 <40 <17 <5 50–120 <40
PT (%)	53%	85%	70–100
Alb (g/l)	24	35	35–50
T-Ch (mmol/l)	1.41	4	3.5–5.2

Alb: Albuminemia; ALP: Alkaline Phosphatase; ALT: Alanine transaminase; AST: Aspartate transaminase; D-Bili: Direct bilirubin; FT4: Hb:Haemoglobin; GGT: Gamma-glutamyl transpeptidase; Plt: Platelets; PT: Prothrombin time; T-Bili: Total bilirubin; T-Ch: Total cholesterol; WBC: White blood cell count.

ECG showed sinus tachycardia and cardiac ultrasound showed no signs of pulmonary hypertension or heart failure.

On the basis of these clinico-biological data, thyroid storm was suspected. The score of the Burch–Wartofsky Point Scale for diagnosis of thyroid storm was 80, confirming our diagnosis (Table 2). Thyroid tests showed low TSH level (<0.01 μIU/ml) and high FT4 level (>76 pmol/l).

**Table 2.** The Burch-Wartofsky point scale of our patient^†^.

Criteria	Points	Patient score
Thermoregulatory dysfunction (temperature °C) • 37.2–37.7 • 37.8–38.3 • 38.4–38.8 • 38.9–39.3 • 39.4–39.9 • ≥40.0	5 10 15 20 25 30	38.9 °C = 20
**Cardiovascular** Tachycardia (beats per min) • 90–109 • 110–119 • 120–129 • 130–139 • ≥140 Atrial fibrillation • Absent • Present	5 10 15 20 25 0 10	130 beats per min = 20
**Congestive heart failure** • Absent • Mild • Moderate • Severe	0 5 10 15	Absent = 0
**Gastrointestinal-hepatic dysfunction** • Absent • Moderate (diarrhea, abdominal pain, nausea/vomiting) • Severe (jaundice)	0 10 20	Jaundice = 20
**Central nervous system disturbance** • Absent • Mild (agitation) • Moderate (delirium, psychosis, lethargy) • Severe (seizure, come)	0 10 20 30	Agitation = 10
**Precipitating event Status** Absent Present	0 10	Treatment withdrawal = 10
**Total score: ≥45 thyroid storm** Impending storm Storm unlikely	25–44 <25	Thyroid storm = 80

† Data taken from [2].

Given the presence of signs of primary adrenal insufficiency (melanoderma and orthostatic hypotension), we perform the short synacthen test (cortisol = 269 nmol/l after stimulation, <550 nmol/l) which confirms the diagnosis. The CT-scan showed normal adrenal glands. The adrenal insufficiency was probably due to an autoimmune pathology. It also explained the imbalance of diabetes with frequent hypoglycemic attacks.

**Figure 1.**
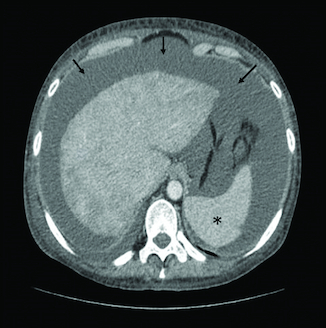
Liver dysmorphia and signs of portal hypertension (splenomegaly [asterisk] and ascites [arrow]) on abdominal CT scan.

Jaunice has been attached to thyroid storm but the cause of the edematous-ascitic syndrome was unclear, particularly in the absence of heart failure. Negative proteinuria excluded the diagnosis of nephrotic syndrome. Despite the absence of diarrhea, malabsorption was suspected because of anemia, hypocholesterolemia and hypoalbuminemia but celiac serology was negative. Jaundice, thrombopenia, hyperbilirubinemia, hypoalbuminemia, hypocholesterolemia and low PT suggested a liver disease. CT-scan showed a dysmorphic liver, portal hypertension signs (splenomegaly and collateral circulation) with permeability of portal and hepatic veins (Figure 1). Upper gastro-intestinal endoscopy revealed small esophageal varices. A decompensated cirrhosis was suggested. Hepatitis B and C serologies were negative. The immunological tests showed: ANA = 1/80, SMA, LKM 1, AMA-M2, SLA and LC-1 were negative. Hepatotoxicity from antithyroid drugs was excluded because the patient has been on treatment for 3 years and the liver was dysmorphic. In view of the autoimmune background and the exudative nature of the ascites, associated lupus was suspected. But there were no other clinical or biological signs and anti-ENA antibodies were negative.

The patient was put on 30 mg hydrocortisone per day with normal sodium diet, insulin, 10 mg methimazole per day, propranolol and preventive anticoagulant. Radioactive iodine was started 15 days later. Two abdominal paracenteses were performed. Despite adrenal insufficiency, the patient received 80 mg furosemide and 100 mg spironolactone per day with an increase of hydrocortisone dose to 40 mg per day. She was discharged 10 days after the radioactive iodine treatment. She did not consult and returned 3 months later. She had stopped diuretics 1 month before. Clinical examination showed no abnormalities. Biological explorations showed a regression of biological disorders (Table 1). Abdominal ultrasonography showed no hepatic dysmorphia, with disappearance of all signs of portal hypertension. Hydrocortisone dose was reduced to 30 mg per day and the patient was asymptomatic 3 months later.

## Discussion

Thyroid storm is a life-threatening exacerbation of hyperthyroidism. Thyroid storm is most commonly associated with underlying Graves' disease, but very rarely with other thyrotoxic disorders such as destructive thyroiditis, toxic multinodular goiter or metastatic thyroid cancer [4,5]. The condition was experienced frequently following acute stress-associated events, pregnancy, non-compliance to anti-thyroid medications and amiodarone use [4,6]. For our patient, the precipitating factor was non-compliance with treatment.

In the absence of specific biological criteria for thyroid storm, the diagnosis is based on clinical findings. A diagnostic score considering the presence and severity of organ dysfunction was developed by Burch and Wartofsky in 1993 [2].

Hepatic injury as part of the multi organ dysfunction due to thyroid storm has been described in several case reports; it ranges from deranged liver enzymes to acute liver failure and fulminant hepatitis [^7-11^].

The pathogenesis of hepatotoxicity in thyroid storm is multifactorial [12,13]. It includes liver congestion as a result of heart failure, ischemic liver lesions due to the increase in hepatic oxygen consumption without a compensatory increase in hepatic blood flow, direct liver toxicity of excess thyroid hormones, through a mitochondrial-dependent pathway, hepatotoxicity of antithyroid drugs in particular thionamides and exacerbation of underlying autoimmune liver disease.

Our patient presented with jaundice, hepatic dysfunction and portal hypertension, in the absence of heart failure which is the most common mechanism of liver damage associated with thyroid storm. Moreover, underlying hepatopathy was unlikely, given the reversible nature of the anomalies and the negativity of the immunological and viral tests. A liver biopsy could not be performed due to the presence of ascites.

The incidence of jaundice in thyroid storms ranged from 17.6–21.3% in a Japanese nationwide survey [3] and severe jaundice was uncommon in patients with thyrotoxicosis in the absence of congestive heart failure or known liver disease. The onset of jaundice and coagulopathy in our patient can be explained by the direct toxic effect of thyroid hormones. In fact, the excess of triiodothyronine (T3) activity can induce hepatocyte apoptosis through a mitochondrial-dependent pathway [14]. In addition, T3 modulate bilirubin metabolism by determining the enzymatic activity of bilirubin-uridine-diphosphate-glucuronosyltransferase (UDP-GT), and excess levels can result in the accumulation of bilirubin precursors.

For portal hypertension, the direct link with thyroid storm was established, retrospectively, after the complete regression concomitant with the normalization of thyroid function. To our knowledge, no similar case associated with thyroid storm has been reported in the literature. The mechanism of portal hypertension was not clear. We hypothesize that it may be due to the transient hypercoagulable state associated with thyroid storm.

In fact, the excess of thyroid hormones modify the coagulation-fibrinolytic balance and it is associated with a hypercoagulable state with an increasing risk of venous thromboembolism [15,16]. This pro-thrombotic state is multifactorial, essentially due to activation of the de Willebrand factor, factor VIII, plasminogen activator inhibitor-1 [16]. This could induce the formation of microthrombi and platelet aggregates in the portal venules, resulting in non-cirrhotic intrahepatic block and the appearance of portal hypertension. The patient was put on anticoagulants during hospitalization because of a potentially thromboembolic risk (thyroid storm and hypoalbuminemia). The complete disappearance of portal hypertension signs supports our hypothesis.

## Conclusion

To sum up, reversible portal hypertension is an exceptional complication of thyroid storm in the absence of congestive heart failure or known liver disease. The etiopathogenic mechanism has yet to be elucidated. Early diagnosis of thyroid storm and appropriate treatment are the cornerstone of the management of this complication.
